# Utility of a Stressed Single Nucleotide Polymorphism (SNP) Real-Time PCR Assay for Rapid Identification of Measles Vaccine Strains in Patient Samples

**DOI:** 10.1128/JCM.00360-18

**Published:** 2018-07-26

**Authors:** Thomas Tran, Renata Kostecki, Michael Catton, Julian Druce

**Affiliations:** a Regional and National Measles Reference Laboratory, Victorian Infectious Diseases Reference Laboratory, Melbourne, Australia; Memorial Sloan Kettering Cancer Center

**Keywords:** measles vaccine, measles vaccine real-time PCR, measles, measles outbreak, measles real-time PCR

## Abstract

Rapid differentiation of wild-type measles virus from measles vaccine strains is crucial during a measles outbreak and in a measles elimination setting. A real-time reverse transcription-PCR (rRT-PCR) for the rapid detection of measles vaccine strains was developed with high specificity and sensitivity equivalent to that of traditional measles genotyping methods. The “stressed” minor groove binder-TaqMan probe design approach achieves specificity to vaccine strains only, without compromising sensitivity. This assay, without requiring sequence genotyping, has proved to be extremely useful in outbreak settings for over 4 years at the Regional Measles Reference Laboratory for the Western Pacific Region.

## Abstract (precis)

Rapid differentiation of wild-type measles virus from measles vaccine strains is crucial during a measles outbreak and in a measles elimination setting. A real-time reverse transcription-PCR (rRT-PCR) for the rapid detection of measles vaccine strains was developed with high specificity and sensitivity equivalent to that of traditional measles genotyping methods.

## INTRODUCTION

Measles is a highly infectious virus, and infection may be associated with significant complications, including encephalitis and potentially fatal pneumonia (1). Measles vaccine coverage of 96 to 99% is required for sufficient herd immunity to prevent outbreaks (2). An increase in vaccine coverage from 73% to 85% worldwide for the first dose of measles-containing vaccine has resulted in a 79% drop in measles deaths between 2000 and 2014, according to the WHO (http://www.who.int/mediacentre/factsheets/fs286/en/). In Australia measles vaccination is provided using either measles-mumps-rubella (MMR) or measles-mumps-rubella-varicella (MMRV) vaccines with the first dose of vaccine given at 12 months of age and a second dose given at 18 months (3). This has achieved measles vaccine coverage close to 94% since 2007 (4). Although the World Health Organization (WHO) has verified the elimination of endemic measles transmission in some countries and regions, including Australia and the Americas (5; see also http://www.who.int/mediacentre/news/notes/2013/measles_20130117/en/), measles continues to occur in these countries, following importation from areas where measles is still prevalent or endemic.

A standard genotyping method for measles viruses has been used since 1998 (6) with the genetic data used to track transmission pathways of measles virus and to document the interruption of endemic measles transmission. Genotyping involves sequencing 450 nucleotides that encode the carboxy-terminal 150 amino acids of the nucleoprotein (N), the minimum WHO requirement for measles genotypic assignment (7). However, this approach, whereby PCR amplicons are electrophoresed on an agarose gel for detection, can potentially result in PCR amplicon contamination, particularly in laboratories that are not equipped with dedicated PCR rooms to minimize contamination events.

In a measles elimination setting, a rapid differentiation of wild-type from vaccine strains is important so that adequate social distancing or quarantine measures can be applied for those with wild-type infections. Standard genotyping may be slow at identifying true wild-type virus cases, and therefore all cases are assumed to be potentially infectious, potentially wasting scarce public health resources on vaccine strain-related cases. Targeted identification of wild-type virus improves efficiency in isolating patients, thereby limiting spread and saving limited human resources and time for contact tracing.

A number of groups have described alternative methods for the laboratory diagnosis of vaccine-associated measles, including loop-mediated isothermal amplification (8), allelic discrimination real-time reverse transcription-PCR (rRT-PCR) (9), RT-PCR-restriction fragment length polymorphism (10), and a more recent real-time PCR using a locked nucleic acid probe (11). However, some of these assays can be complex or slow.

Here, we describe the first 4 years of use of a simple and rapid measles vaccine TaqMan rRT-PCR that has been designed to span a variable region of the N gene, with a probe that matches vaccine strains but not circulating genotypes. This assay, when used in conjunction with a measles screening assay, can easily be implemented into any standard diagnostic laboratory for the rapid, sensitive, and specific detection of measles vaccine-associated cases.

## MATERIALS AND METHODS

### Measles samples and reference sequences.

An MMR vaccine containing the Moraten measles vaccine strain (MMR II; CSL Limited/Merck and Co., Inc.) was obtained for validation of the measles vaccine rRT-PCR. A log dilution series of the cDNA, generated from the measles-containing vaccine, was prepared and tested.

Confirmation of specificity involved analyzing measles genotype reference sequences (GenBank accession numbers U01987, U01998, U01994, L46753, AJ232203, AY043459, M89921, X84872, D01005, U64582, U01977, U01976, L46758, AF079555, L46750, AF243450, AY037020, AF280803, AF481485, AY923185, GU440571, X84879, X84865, U01974, AF171232, AY184217, AF045212, AF045217, AF266287, AF266291, AF266289, AF266290, AF266286, U03661, FJ416068, FJ416067, and U03650), testing clinical samples previously genotyped as measles vaccine strain and measles wild-type strains (accession numbers DQ398077, KM283208, KJ690779, AF481488, KF433081, AF481482, AF481493, KT835079, KF938655, and KF591386) (see Table 4), and other viruses, including those from the Paramyxoviridae family (mumps virus, parainfluenza viruses, respiratory syncytial virus, and human metapneumovirus).

Parallel testing of the measles rRT-PCR and measles vaccine rRT-PCR was conducted on 3,114 clinical samples (throat swabs, pooled nose and throat swabs, urine, nasopharyngeal aspirate, and blood), for which measles rRT-PCR was requested between January 2014 and November 2017.

### Viral RNA extraction and cDNA synthesis.

Clinical specimens for molecular viral studies (including of measles virus) were extracted using a QIAamp viral RNA minikit (Qiagen), as previously described (12). Briefly, an RNA virus internal control (bovine viral diarrhea virus [BVDV]) was spiked into every sample prior to nucleic acid extraction to control the extraction, reverse transcription, and real-time RT-PCR steps (13). Extracted RNA was converted to cDNA by reverse transcription with random hexamers using the SensiFAST cDNA synthesis kit from Bioline (Bioline Reagents Ltd., UK) according to the manufacturer's instructions. cDNA was used immediately in the real-time PCR or stored at −20°C.

### Real-time PCR assays.

**FIG 1:**
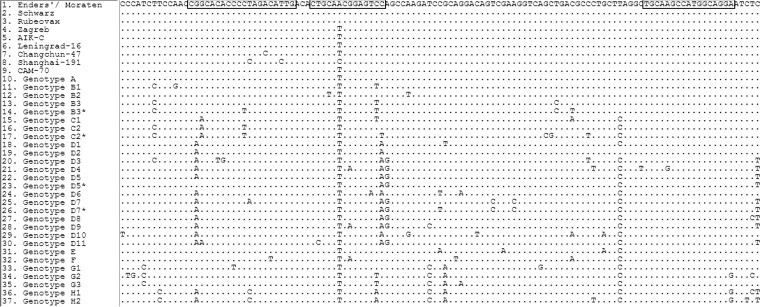
Primer and probe design for the measles vaccine rRT-PCR targeting the N gene. The primer and probe location and sequence used in the measles vaccine real-time PCR are boxed. Strains 1 to 9 are vaccine strains. Edmonston wild-type genotype A is represented as strain 10. Strains 11 to 37 are WHO wild-type reference genotype strains. An asterisk (*) denotes an alternative reference strain. Identical nucleotides compared to the Edmonston-derived vaccine strains (Enders/Moraten, Schwarz, and Rubeovax) are represented with dots. Nucleotide differences compared to Edmonston-derived vaccine strains are shown and are indicated as the dNTP base.

The measles vaccine rRT-PCR was run concurrently with or following a measles rRT-PCR screening assay, as previously described (14). Primers and the minor groove-binding (MGB) probe for the measles vaccine rRT-PCR (targeting the most conserved region for genotype A with variability from other genotypes of the measles N gene) (Fig. 1) were designed using Primer Express Software 3.0 (Applied Biosystems, Foster City, CA). The optimal probe length derived from Primer Express was shortened by 2 nucleotides from the software parameters, reducing the *T_m_* by 3°C.

Briefly, 3 μl of cDNA was added to a final 20-μl reaction mix containing primers and probe (Table 1), with a final concentration of 0.9 μM and 0.2 μM each primer and probe, respectively. A separate rRT-PCR for the internal control was performed for each sample in a separate well, and both positive and no-template controls were included with each PCR run. Thermal cycling and real-time PCR analysis for all assays were performed on the ABI 7500 FAST real-time PCR system (Applied Biosystems, Foster City, CA) with the following thermal cycling profile: 95°C for 20 s, followed by 45 PCR cycles of 95°C for 3 s and 60°C for 30 s.

**TABLE 1:** Primers and probes for measles vaccine rRT-PCR, measles rRT-PCR, and measles genotyping RT-PCR

Method	Primer or probe^*a*^	5′–3′ nucleotide sequence
Measles vaccine rRT-PCR	MeVAvac-F	CGGCACACCCCTAGACATTG
	MeVAvac-R	TCCTGCCATGGCTTGCA
	MeVAvac-P	FAM-CTGCAACGGAGTCC-MGBNFQ
Measles rRT-PCR	MeV-F	TGGCATCYGAACTCGGTATCAC
	MeV-R	TGTCCTCAGTAGTATGCATTGCAA
	MeV-P	FAM-CCGAGGATGCAAGGCTWGTTTCAGA-TAMRA
Measles genotyping RT-PCR	MeV216-F	TGGAGCTATGCCATGGGAGT
	MeV214-R	TAACAATGATGGAGGGTAGG

a F, forward primer; R, reverse primer; P, probe.

### Measles genotyping PCR and sequencing.

Measles genotyping PCR was performed, using a modified version of the WHO protocol (15), on measles rRT-PCR-positive samples or when there was a measles rRT-PCR-positive/measles vaccine rRT-PCR-negative result. The modification involved a second round of measles genotyping PCR, using the primers MeV216 and MeV214 (16). The PCR product targeting 450 nucleotides of the carboxyl end of N (N-450) was sequenced in the forward and reverse directions, as previously described (15).

## RESULTS

### Sensitivity, specificity, and performance of rRT-PCR assays.

**FIG 2:**
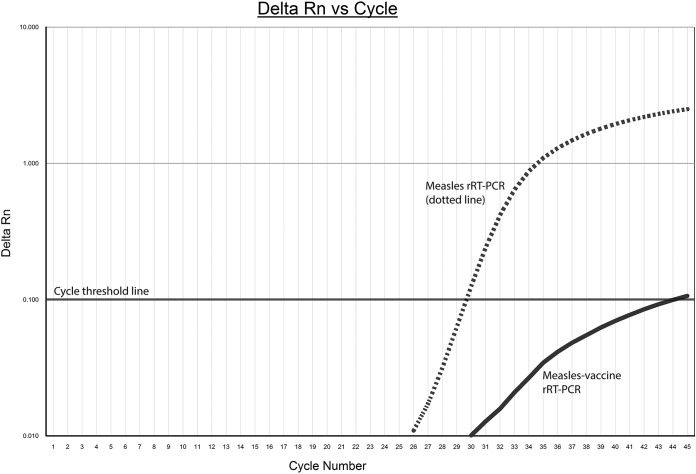
Amplification profiles of wild-type Edmonston strain (genotype A) measles on measles rRT-PCR and measles vaccine rRT-PCR. The dashed line shows the amplification plot generated by the measles rRT-PCR, and the solid line shows the deformed amplification plot generated by measles vaccine real-time PCR. The solid horizontal line is the cycle threshold line from which amplification curves cross to generate *C_T_* values.

The limits of detection (LODs) of the measles rRT-PCR, measles genotyping PCR, and measles vaccine rRT-PCR were measured using serial 10-fold dilutions of the MMR II vaccine. The LODs for all three assays were equivalent, reliably detecting vaccine virus in 20 replicates at 10^−3^ dilution (Table 2). Measles RNA detection at 10^−4^ dilution was less reproducible for all three assays and was not detected at 10^−5^ dilution for all assays. The measles vaccine rRT-PCR was specific to Edmonston-derived vaccine strains and less specific to the wild-type Edmonston strain genotype A (Table 3), with the latter weakly reacting in this assay with a deformed amplification curve compared to that of the measles rRT-PCR (Fig. 2). No positive reactions were recorded against other genotypes assessed, including those with high burdens of virus (cycle threshold, ≤25) (Table 4). Other paramyxoviruses evaluated also tested negative with the measles vaccine rRT-PCR (results not shown).

**TABLE 2:**
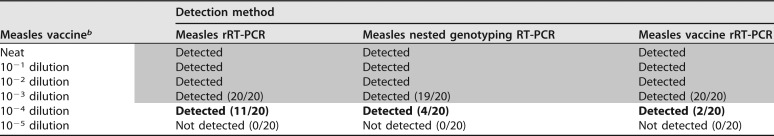
Sensitivity of detection for vaccine strain^*a*^

a Serial log10 dilutions of cDNA from measles vaccine (MMR II-CSL) used to assess the limit of detection for all PCR assays. Dilutions (10^−3^ to 10^−5^) of the measles vaccine strain were tested in 20 replicates, with results shown in parentheses. Shaded boxes indicate reproducible detection of measles virus RNA. Nonreproducible detection of measles virus RNA shown in boldface. Unshaded boxes indicate that measles virus RNA was not detected.

^*b*^MMR II (CSL Limited/Merck and Co., Inc.).

**TABLE 3:** Comparison of specificity and sensitivity of wild-type Edmonston strain (genotype A) detection in the measles rRT-PCR and the measles vaccine rRT-PCR

Measles Edmonston wild-type strain	Viral RNA detection results by^*a*^:	
	Measles rRT-PCR (*C_T_* value)	Measles vaccine rRT-PCR (*C_T_* value)
Neat	Detected (30)	Detected (44)
10^−1^ dilution	Detected (33)	Not detected
10^−2^ dilution	Detected (37)	Not detected
10^−3^ dilution	Not detected	Not detected
10^−4^ dilution	Not detected	Not detected

a Real-time PCR cycle threshold (*C_T_*) values are provided in parentheses.

**TABLE 4:** Specificity of the measles vaccine rRT-PCR to vaccine strain, wild-type Edmonston strain (genotype A) and other circulating wild-type genotypes of measles virus

Measles genotype	Detection results by:^*a*^	
	Measles rRT-PCR (*C_T_* value)	Measles vaccine rRT-PCR (*C_T_* value)
A-vaccine	Detected (25)	Detected (25)
A-wild type	Detected (25)	Detected (41)^*b*^
B3	Detected (23)	Not detected
D3	Detected (21)	Not detected
D4	Detected (24)	Not detected
D5	Detected (21)	Not detected
D7	Detected (23)	Not detected
D8	Detected (19)	Not detected
D9	Detected (20)	Not detected
G3	Detected (25)	Not detected
H1	Detected (21)	Not detected
H2	Detected (17)	Not detected

a Real-time PCR cycle threshold (*C_T_*) values are provided in parentheses.

b Amplification curve exhibited with a deformed amplification curve (see Fig. 2).

### Clinical sample validation.

**FIG 3:**
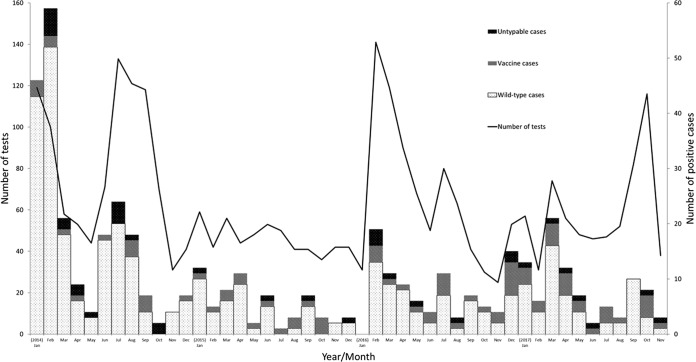
Monthly distribution of measles PCR testing and measles genotyped cases from January 2014 to November 2017.

From January 2014 to November 2017, 3,114 samples were prospectively screened for measles RNA by the measles rRT-PCR. Of these, 624 (20.3%) from 471 measles cases had measles RNA detected. A genotype was achieved for 434 (92%) of these cases. The remaining 37 cases had a measles RNA copy number (*C_T_*, >37) that was too low to determine the genotype (Table 5 and Fig. 3).

**TABLE 5:** No. of measles cases and measles genotyped cases from prospective parallel testing of clinical specimens from January 2014 to November 2017

No. of cases	Time period	
	January 2014–September 2015	October 2015–November 2017
Measles cases	299	172
Measles cases, genotyped/untypeable	272/27	162/10
Measles vaccine case investigations	46	105
Measles vaccine case investigations positive by measles rRT-PCR/measles vaccine rRT-PCR/measles genotyping PCR	27/27/25	40/40/NA^*a*^

a NA, measles genotyping PCR not performed.

There were 151 investigations of measles vaccine-associated cases during the evaluation period (Table 5). Between January 2014 and September 2015, 46 cases clinically assumed to be vaccine associated were tested concurrently with the measles vaccine rRT-PCR and genotyping PCR. Of these, 25 cases were positive for measles vaccine by both the measles vaccine rRT-PCR and the genotyping PCR, and 19 were negative for the vaccine strain by both methods. There were 2 discordant results that were measles vaccine rRT-PCR-positive/genotype PCR-negative. Of the 19 measles vaccine case investigations that were negative by both methods, 6 were subsequently shown to be genotype B3, 1 was genotype D8, and 12 were negative by both measles rRT-PCR and vaccine rRT-PCR. From October 2015 to November 2017, there were 105 investigations of measles vaccine-associated cases where only the measles vaccine rRT-PCR was used. Of these, 40 cases were positive by both the measles rRT-PCR and measles vaccine rRT-PCR, and 60 were negative by both measles rRT-PCR and measles vaccine rRT-PCR. There were 5 cases of measles that were rRT-PCR-positive/measles vaccine rRT-PCR-negative, of which 3 were genotyped as measles D8, and 2 were genotype PCR-negative due to a high cycle threshold (*C_T_*, >40). In addition, retrospective screening of 10 measles samples previously sequenced as vaccine-associated measles by the vaccine rRT-PCR resulted in no discordance (results not shown).

## DISCUSSION

Here, we describe a measles vaccine rRT-PCR incorporating an MGB TaqMan probe that had its performance tolerance deliberately stressed, providing the capacity to quickly differentiate measles vaccine strains from the wild-type virus. This allows health professionals to focus on contact tracing and quarantine/isolation of wild-type measles cases, which is crucial in reducing onward transmission during measles importations.

This method also removes the time delay and potential amplicon contamination associated with sequencing. Other groups have previously reported methods for detecting measles vaccine strains. Nakayama et al. described a simple, loop-mediated isothermal amplification method that differentiates measles vaccine strains from wild-type strains, based on which primer set reaches the amplification threshold fastest (8). However, this method may have difficulties discriminating wild-type C1, C2, B2, and B3 from vaccine strains. An RT-PCR restriction fragment length polymorphism method from Zhou et al. requires an additional step of enzyme digestion for measles strain differentiation (10). Other assays by Xu et al. and Roy et al. are more similar to the one herein described with the former reporting further additional validation requirements to determine sensitivity and specificity as only 8 clinical samples were assessed (9, 11). Alternatively, the more recently published method by Roy et al. uses a locked nucleic acid probe to achieve a higher annealing temperature which enables shorter probe lengths to be utilized, similar to MGB probe chemistry (11).

The design of measles vaccine-specific primers and probe was challenging, due to the complex nucleotide mixtures that differentiate genotypes in the N gene. The region chosen for the probe spanned a variable region with the maximal conservation to measles vaccine strains used in Australia (Moraten and Schwarz). For most genotypes, there were 2 or more single-nucleotide polymorphisms (SNPs) across the probe region, reducing the likelihood of reaction. To reduce the risk of probe-binding tolerance to other genotypes where there is a 1-nucleotide difference, the optimal length of the probe was shortened by 2 nucleotides to stress the reaction kinetics of probe binding under normal cycling conditions. This limited the reactive range to other vaccine strains not administered in Australia (Zagreb, AIK-C, Leningrad-16, Changchun-47, Shanghai-191, and CAM-70) and to genotype A, C2, E, and G1, all of which are no longer circulating as wild-type viruses. Reactivity to these other vaccine strains, most notably to Edmonston Zagreb (due to its use in some countries), is likely to behave similarly to reactivity to wild-type Edmonston strain with an altered amplification curve. To compensate, specificity to vaccine strains was enhanced, with the forward primer and the reverse primer spanning some nucleotide variation for selected genotypes without incorporating mixed nucleotides or degenerate primers. The stressed probe design allowed the probe to span a signature single nucleotide polymorphism (SNP) in a region with low intermittent background nucleotide sequence variability and intolerance to any mismatch. Furthermore, the built-in compromise of primer binding to nonvaccine strains facilitates greater specificity to vaccine strains without cross-reaction with other circulating measles genotypes.

A limitation of the evaluation was not being able to check the specificity performance of the design against all 24 known measles genotypes. Despite the lack of positive-control material for this assessment, alignment of all WHO measles reference strains and mapping of the primer probe sequence suggest possible cross-reactivity with only 4 wild-type genotypes (A, C2, E, and G1), all of which are currently considered inactive (17). Any cross-reactivity with these genotypes would likely be seen as a deformed amplification profile with a significant increase in *C_T_* value compared to that obtained in the measles rRT-PCR. This would suggest some nucleotide mismatch to nonvaccine strains in the unfavorable PCR design. If the measles vaccine rRT-PCR fails to detect at the LOD (or has reduced sensitivity or generates a deformed amplification curve), these discrepancies will be determined by routine genotyping of the N-450 PCR product where possible.

Prospective testing of clinical specimens throughout the evaluation period using the measles vaccine rRT-PCR detected additional vaccine-associated cases that could not be verified by genotyping. These were unlikely to have been false vaccine-positive results because (i) they were positive by the measles screening test and (ii) follow-up of these cases indicated that they had been recently vaccinated prior to specimen collection and that cases were only mildly symptomatic. Genotyping of measles-positive rRT-PCR/measles vaccine-negative rRT-PCR samples did not identify any additional measles vaccine cases.

In conclusion, this SNP assay performs reliably against a background of various measles genotype-specific SNPs to rapidly determine whether a measles-positive sample is vaccine associated or wild type. It has been incorporated into our standard testing algorithm, along with concurrent diagnostic and vaccine rRT-PCR, when vaccination is known to have been performed. This approach rapidly differentiates wild-type cases, which are the focus of public health containment and quarantine measures.
